# Poly[diaqua­[3,5-bis­(trifluoro­meth­yl)pyrazolido]potassium]

**DOI:** 10.1107/S1600536810027133

**Published:** 2010-07-14

**Authors:** Hien Ngoc Phan, Hans-Wolfram Lerner, Michael Bolte

**Affiliations:** a Institut für Anorganische Chemie, J. W. Goethe-Universität Frankfurt, Max-von-Laue-Strasse 7, 60438 Frankfurt/Main, Germany

## Abstract

The asymmetric unit of the title compound, [K(C_5_HF_6_N_2_)(H_2_O)_2_]_*n*_, is composed of two 3,5-bis­(trifluoro­meth­yl)pyrazol­ide anions, two potassium cations and four water mol­ecules. The water mol­ecules and 3,5-bis­(trifluoro­meth­yl)pyrazolide anions act as bridges between the potassium cations. Each potassium cation is surrounded by four O atoms [K—O = 2.705 (3)–2.767 (3) Å] and four F atoms [K—F = 2.870 (7)–3.215 (13) Å]. The water mol­ecules and the 3,5-bis­(trifluoro­meth­yl)pyrazolide anions are connected by O—H⋯N hydrogen bonds, forming layers in the *ab* plane. All –CF_3_ groups show rotational disorder between two orientations each.

## Related literature

For related literature on pyrazolides, see: Bieller *et al.* (2006 ▶).
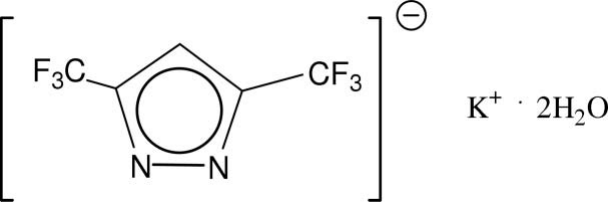


## Experimental

### 

#### Crystal data

[K(C_5_HF_6_N_2_)(H_2_O)_2_]
*M* *_r_* = 278.21
Triclinic, 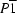

*a* = 9.7453 (9) Å
*b* = 9.8179 (10) Å
*c* = 12.5243 (14) Å
α = 67.756 (8)°
β = 78.178 (8)°
γ = 88.758 (8)°
*V* = 1083.53 (19) Å^3^
*Z* = 4
Mo *K*α radiation
μ = 0.56 mm^−1^
*T* = 173 K
0.41 × 0.40 × 0.38 mm

#### Data collection

Stoe IPDS II two-circle diffractometer
Absorption correction: multi-scan (*MULABS*; Spek, 2009 ▶; Blessing, 1995 ▶) *T*_min_ = 0.802, *T*_max_ = 0.815
11313 measured reflections
4027 independent reflections
3316 reflections with *I* > 2σ(*I*)
*R*_int_ = 0.039

#### Refinement

*R*[*F* ^2^ > 2σ(*F* ^2^)] = 0.049
*wR*(*F* ^2^) = 0.124
*S* = 1.13
4027 reflections
425 parameters
704 restraints
H atoms treated by a mixture of independent and constrained refinement
Δρ_max_ = 0.55 e Å^−3^
Δρ_min_ = −0.34 e Å^−3^

### 

Data collection: *X-AREA* (Stoe & Cie, 2001 ▶); cell refinement: *X-AREA*; data reduction: *X-AREA*; program(s) used to solve structure: *SHELXS97* (Sheldrick, 2008 ▶); program(s) used to refine structure: *SHELXL97* (Sheldrick, 2008 ▶); molecular graphics: *XP* (Sheldrick, 2008 ▶) and *Mercury* (Macrae *et al.*, 2006 ▶); software used to prepare material for publication: *SHELXL97*.

## Supplementary Material

Crystal structure: contains datablocks I, global. DOI: 10.1107/S1600536810027133/cv2744sup1.cif

Structure factors: contains datablocks I. DOI: 10.1107/S1600536810027133/cv2744Isup2.hkl

Additional supplementary materials:  crystallographic information; 3D view; checkCIF report

## Figures and Tables

**Table 1:** Hydrogen-bond geometry (Å, °)

*D*—H⋯*A*	*D*—H	H⋯*A*	*D*⋯*A*	*D*—H⋯*A*
O1—H1*A*⋯N11^i^	0.82 (2)	2.10 (2)	2.906 (4)	167 (4)
O1—H1*B*⋯N12^ii^	0.82 (2)	2.09 (2)	2.896 (4)	168 (4)
O2—H2*A*⋯N2	0.82 (2)	2.12 (1)	2.929 (4)	173 (5)
O2—H2*B*⋯N2^iii^	0.81 (2)	2.07 (1)	2.879 (4)	173 (5)
O3—H3*A*⋯N12	0.82 (2)	2.05 (1)	2.868 (4)	173 (4)
O3—H3*B*⋯N1	0.82 (2)	2.09 (1)	2.910 (4)	177 (4)
O4—H4*A*⋯N11^i^	0.82 (2)	2.08 (1)	2.891 (4)	175 (4)
O4—H4*B*⋯N1^i^	0.82 (2)	2.07 (1)	2.884 (4)	174 (4)

Symmetry codes: (i) 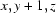; (ii) 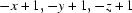; (iii) 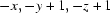.

**Fig. 1.**
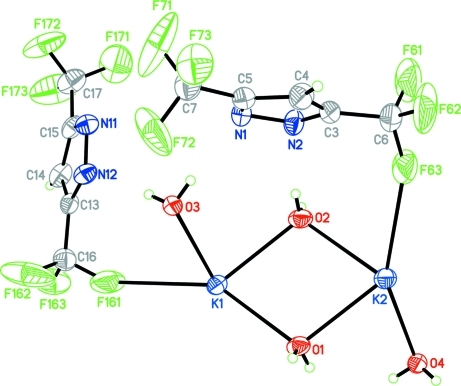
Perspective view of the asymmetric unit of the title compound with the atom numbering scheme; displacement ellipsoids are at the 50% probability level; H atoms are drawn as small spheres of arbitrary radii. Only major components of the disordered CF3 groups are shown.

**Fig. 2.**
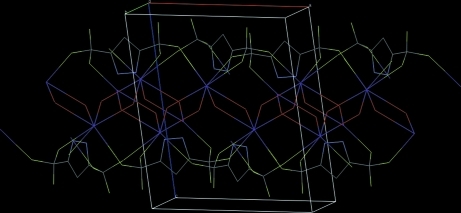
A portion of the packing diagram showing the layer structure. Only major components of the disordered CF3 groups are shown.

## supplementary crystallographic information

### Comment

Recently we have reported the structures of pyrazolides which possess substituents in a 3- or 4-position (Bieller *et al.*, 2006). In this paper we report the synthesis and the crystal structure of the 3,5-substituted potassium pyrazolide [K(H_2_O)_2_][3,5-(CF_3_)_2_C_3_HN_2_] (I). The starting matrial for the synthesis of (I), 3,5-bis(trifluoromethyl)pyrazole, was prepared from hexafluoroacetylacetone CF_3_COCH_2_COCF_3_ and hydrazine hydrate N_2_H_4_^.^H_2_O. By a following reaction of one equivalent of KH and one equivalent of 3,5-bis(trifluoromethyl)pyrazole in ethanol the potassium pyrazolide (I) was accessible in nearly quantitive yield.

The asymmetric unit of the title compound is composed of two 3,5-bis(trifluoromethyl)pyrazolide anions, two potassium cations and four water molecules. The water molecules and 3,5-bis(trifluoromethyl)pyrazolide anions act as bridges between the potassium cations. Each potassium cation is bonded to four O atoms and four F atoms. The K—O distances range from 2.705 (3)Å (K2—O4) to 2.767 (3)Å (K1—O3) and the K—F distances range from 2.870 (7)Å (K2—F162) to 3.215 (13)Å (K2—F17'). The water molecules and the 3,5-bis(trifluoromethyl)pyrazolide anions are connected by O—H···N hydrogen bonds forming layers in the *ab* plane The CF_3_ groups show rotational disorder.

### Experimental

3,5-Bis(trifluoromethyl)pyrazole: Hydrazine hydrate N_2_H_4_^.^H_2_O (2.65 g, 52.87 mmol) and hexafluoroacetylacetone CF_3_COCH_2_COCF_3_ (10.0 g, 6.8 ml, 48.06 mmol) were combined in ethanol (100 ml) under ambient conditions, forming a clear solution. The solution was heated under reflux for 5 h. After addition of a small amount of Na_2_SO_4_ the reaction mixture was heated again for 6 h. After removing the solvent pure, colorless 3,5-bis(trifluoromethyl)pyrazole was obtained by sublimation (373 K, normal pressure) in 79% yield.

Potassium 3,5-bis(trifluoromethyl)pyrazolide (I): To a slurry of KH (138 mg, 3.44 mmol) in 15 ml of tetrahydrofuran was added a solution of 3,5-bis(trifluoromethyl)pyrazole (820 mg, 4.02 mmol) in 15 ml tetrahydrofuran at 273 K. The resulting solution was allowed to warm up to room temperature. After removing the solvent *in vacuo* the obtained residue was recrystallized from wet tetrahydrofuran (yield 92%).

### Refinement

H atoms were found in a difference map but those bonded to C were refined with fixed individual displacement parameters [U_iso_(H) = 1.2 *U*_eq_(C)] using a riding model with C—H = 0.95 Å. H atoms bonded to O were refined with U_iso_(H) = 1.2 *U*_eq_(O) and a distance restraint of 0.82 (1) Å. The four trifluoromethyl groups are disordered over two positions each with site occupation factors of 0.69 (4), 0.52 (3), 0.57 (3) and 0.69 (3) for the major occupied sites. The C—F bond lengths and the equivalent F···F distances were restrained to be equal with an effective s.u. of 0.02 Å. The anisotropic displacement ellipsoids of the F atoms were restrained to an isotropic behaviour.

### Crystal data

**Table:**

[K(C_5_HF_6_N_2_)(H_2_O)_2_]	*Z* = 4
*M**_r_* = 278.21	*F*(000) = 552
Triclinic, *P*1	*D*_x_ = 1.705 Mg m^−^^3^
Hall symbol: -P 1	Mo *K*α radiation, λ = 0.71073 Å
*a* = 9.7453 (9) Å	Cell parameters from 10340 reflections
*b* = 9.8179 (10) Å	θ = 3.6–25.7°
*c* = 12.5243 (14) Å	µ = 0.56 mm^−^^1^
α = 67.756 (8)°	*T* = 173 K
β = 78.178 (8)°	Block, colourless
γ = 88.758 (8)°	0.41 × 0.40 × 0.38 mm
*V* = 1083.53 (19) Å^3^	

### Data collection

**Table:**

Stoe IPDS II two-circle diffractometer	4027 independent reflections
Radiation source: fine-focus sealed tube	3316 reflections with *I* > 2σ(*I*)
graphite	*R*_int_ = 0.039
ω scans	θ_max_ = 25.6°, θ_min_ = 3.6°
Absorption correction: multi-scan (*MULABS*; Spek, 2009; Blessing, 1995)	*h* = −11→11
*T*_min_ = 0.802, *T*_max_ = 0.815	*k* = −11→11
11313 measured reflections	*l* = −15→15

### Refinement

**Table:**

Refinement on *F*^2^	Primary atom site location: structure-invariant direct methods
Least-squares matrix: full	Secondary atom site location: difference Fourier map
*R*[*F*^2^ > 2σ(*F*^2^)] = 0.049	Hydrogen site location: inferred from neighbouring sites
*wR*(*F*^2^) = 0.124	H atoms treated by a mixture of independent and constrained refinement
*S* = 1.13	*w* = 1/[σ^2^(*F*_o_^2^) + (0.0432*P*)^2^ + 1.5007*P*] where *P* = (*F*_o_^2^ + 2*F*_c_^2^)/3
4027 reflections	(Δ/σ)_max_ < 0.001
425 parameters	Δρ_max_ = 0.55 e Å^−^^3^
704 restraints	Δρ_min_ = −0.34 e Å^−^^3^

### Special details

**Table:**

Geometry. All e.s.d.'s (except the e.s.d. in the dihedral angle between two l.s. planes) are estimated using the full covariance matrix. The cell e.s.d.'s are taken into account individually in the estimation of e.s.d.'s in distances, angles and torsion angles; correlations between e.s.d.'s in cell parameters are only used when they are defined by crystal symmetry. An approximate (isotropic) treatment of cell e.s.d.'s is used for estimating e.s.d.'s involving l.s. planes.
Refinement. Refinement of *F*^2^ against ALL reflections. The weighted *R*-factor *wR* and goodness of fit *S* are based on *F*^2^, conventional *R*-factors *R* are based on *F*, with *F* set to zero for negative *F*^2^. The threshold expression of *F*^2^ > σ(*F*^2^) is used only for calculating *R*-factors(gt) *etc*. and is not relevant to the choice of reflections for refinement. *R*-factors based on *F*^2^ are statistically about twice as large as those based on *F*, and *R*- factors based on ALL data will be even larger.

### Fractional atomic coordinates and isotropic or equivalent isotropic displacement parameters (Å^2^)

**Table:**

	*x*	*y*	*z*	*U*_iso_*/*U*_eq_	Occ. (<1)
K1	0.39698 (8)	0.67416 (8)	0.37909 (7)	0.0306 (2)	
K2	0.08656 (8)	0.81180 (8)	0.61839 (8)	0.0317 (2)	
O1	0.3599 (3)	0.8616 (3)	0.4958 (2)	0.0302 (6)	
H1A	0.368 (5)	0.9497 (17)	0.455 (3)	0.036*	
H1B	0.410 (4)	0.841 (5)	0.544 (3)	0.036*	
O2	0.1232 (3)	0.6242 (3)	0.5002 (3)	0.0341 (6)	
H2A	0.104 (5)	0.539 (2)	0.547 (3)	0.041*	
H2B	0.075 (4)	0.630 (5)	0.453 (3)	0.041*	
O3	0.3856 (3)	0.3788 (3)	0.5015 (2)	0.0298 (6)	
H3A	0.407 (4)	0.337 (4)	0.456 (3)	0.036*	
H3B	0.314 (3)	0.335 (4)	0.549 (3)	0.036*	
O4	0.1246 (3)	1.1129 (3)	0.4996 (3)	0.0322 (6)	
H4A	0.199 (3)	1.128 (5)	0.452 (3)	0.039*	
H4B	0.128 (5)	1.152 (4)	0.546 (3)	0.039*	
N1	0.1337 (3)	0.2318 (3)	0.6765 (3)	0.0326 (7)	
N2	0.0336 (3)	0.3299 (3)	0.6808 (3)	0.0338 (7)	
C3	−0.0136 (4)	0.3079 (4)	0.7954 (4)	0.0359 (9)	
C4	0.0550 (4)	0.1969 (4)	0.8683 (4)	0.0378 (9)	
H4	0.0428	0.1606	0.9518	0.045*	
C5	0.1458 (4)	0.1523 (4)	0.7882 (3)	0.0306 (8)	
C6	−0.1252 (5)	0.3993 (5)	0.8265 (4)	0.0512 (12)	
C7	0.2477 (4)	0.0352 (5)	0.8098 (4)	0.0435 (10)	
N11	0.3789 (3)	0.1607 (3)	0.3208 (3)	0.0332 (7)	
N12	0.4741 (3)	0.2560 (3)	0.3258 (3)	0.0319 (7)	
C13	0.5380 (4)	0.3394 (4)	0.2138 (3)	0.0287 (8)	
C14	0.4871 (4)	0.3002 (4)	0.1328 (3)	0.0356 (9)	
H14	0.5142	0.3398	0.0491	0.043*	
C15	0.3864 (4)	0.1881 (4)	0.2058 (3)	0.0339 (8)	
C16	0.6470 (4)	0.4559 (4)	0.1921 (3)	0.0367 (9)	
C17	0.2902 (5)	0.1001 (5)	0.1750 (4)	0.0492 (11)	
F61	−0.2511 (7)	0.3608 (16)	0.8164 (17)	0.083 (3)	0.69 (4)
F62	−0.141 (2)	0.3797 (16)	0.9412 (6)	0.083 (3)	0.69 (4)
F63	−0.1015 (14)	0.5433 (9)	0.7639 (14)	0.072 (3)	0.69 (4)
F61'	−0.233 (2)	0.413 (3)	0.768 (3)	0.064 (6)	0.31 (4)
F62'	−0.188 (3)	0.354 (2)	0.9391 (12)	0.062 (5)	0.31 (4)
F63'	−0.076 (3)	0.5390 (19)	0.796 (2)	0.053 (5)	0.31 (4)
F71	0.259 (2)	−0.040 (2)	0.7424 (16)	0.073 (4)	0.48 (3)
F72	0.3831 (9)	0.0999 (13)	0.7868 (15)	0.063 (4)	0.48 (3)
F73	0.2264 (18)	−0.053 (2)	0.9224 (9)	0.053 (3)	0.48 (3)
F71'	0.1992 (18)	−0.0831 (11)	0.7881 (14)	0.062 (3)	0.52 (3)
F72'	0.3724 (13)	0.0738 (14)	0.7403 (16)	0.085 (5)	0.52 (3)
F73'	0.2595 (19)	−0.0300 (18)	0.9222 (9)	0.048 (3)	0.52 (3)
F161	0.6176 (13)	0.5317 (19)	0.2597 (15)	0.074 (4)	0.56 (3)
F162	0.7721 (8)	0.3939 (10)	0.2141 (13)	0.060 (3)	0.56 (3)
F163	0.6807 (15)	0.5469 (16)	0.0798 (8)	0.056 (3)	0.56 (3)
F16'	0.5838 (12)	0.5759 (11)	0.2114 (14)	0.052 (3)	0.44 (3)
F16"	0.737 (2)	0.4186 (15)	0.2627 (18)	0.077 (5)	0.44 (3)
F16*	0.7155 (19)	0.5199 (18)	0.0808 (10)	0.051 (3)	0.44 (3)
F171	0.1522 (14)	0.085 (3)	0.240 (3)	0.072 (6)	0.31 (3)
F172	0.327 (3)	−0.0401 (16)	0.204 (2)	0.054 (5)	0.31 (3)
F173	0.276 (3)	0.147 (2)	0.0655 (13)	0.069 (5)	0.31 (3)
F17'	0.1599 (10)	0.1422 (15)	0.1863 (15)	0.083 (3)	0.69 (3)
F17"	0.2852 (16)	−0.0440 (8)	0.2354 (12)	0.071 (3)	0.69 (3)
F17*	0.3321 (18)	0.1236 (12)	0.0586 (5)	0.083 (3)	0.69 (3)

### Atomic displacement parameters (Å^2^)

**Table:**

	*U*^11^	*U*^22^	*U*^33^	*U*^12^	*U*^13^	*U*^23^
K1	0.0294 (4)	0.0256 (4)	0.0395 (5)	0.0024 (3)	−0.0082 (3)	−0.0150 (3)
K2	0.0276 (4)	0.0264 (4)	0.0422 (5)	0.0031 (3)	−0.0106 (3)	−0.0126 (4)
O1	0.0291 (13)	0.0230 (12)	0.0404 (16)	0.0028 (10)	−0.0151 (11)	−0.0101 (12)
O2	0.0285 (14)	0.0260 (13)	0.0490 (18)	0.0002 (11)	−0.0149 (12)	−0.0122 (13)
O3	0.0251 (13)	0.0299 (14)	0.0362 (16)	−0.0018 (10)	−0.0002 (11)	−0.0177 (12)
O4	0.0236 (13)	0.0334 (14)	0.0474 (18)	0.0031 (11)	−0.0082 (12)	−0.0238 (13)
N1	0.0297 (16)	0.0310 (16)	0.0386 (19)	0.0058 (13)	−0.0028 (13)	−0.0176 (15)
N2	0.0324 (16)	0.0315 (17)	0.0379 (19)	0.0099 (13)	−0.0064 (14)	−0.0147 (15)
C3	0.034 (2)	0.0299 (19)	0.038 (2)	−0.0014 (16)	0.0058 (16)	−0.0143 (17)
C4	0.047 (2)	0.034 (2)	0.027 (2)	0.0008 (17)	0.0003 (17)	−0.0107 (17)
C5	0.0298 (18)	0.0280 (19)	0.032 (2)	0.0000 (14)	−0.0046 (15)	−0.0102 (16)
C6	0.050 (3)	0.040 (2)	0.058 (3)	0.005 (2)	0.009 (2)	−0.022 (2)
C7	0.049 (3)	0.038 (2)	0.037 (2)	0.0113 (19)	−0.0088 (19)	−0.0077 (19)
N11	0.0379 (17)	0.0306 (16)	0.0335 (18)	−0.0010 (13)	−0.0097 (14)	−0.0134 (14)
N12	0.0391 (17)	0.0276 (16)	0.0333 (18)	0.0013 (13)	−0.0119 (14)	−0.0141 (14)
C13	0.0320 (18)	0.0254 (18)	0.0281 (19)	0.0057 (14)	−0.0059 (15)	−0.0101 (15)
C14	0.052 (2)	0.031 (2)	0.0240 (19)	0.0079 (17)	−0.0116 (17)	−0.0091 (16)
C15	0.047 (2)	0.0271 (19)	0.032 (2)	0.0101 (16)	−0.0173 (17)	−0.0121 (16)
C16	0.037 (2)	0.034 (2)	0.033 (2)	−0.0007 (16)	−0.0033 (17)	−0.0082 (18)
C17	0.065 (3)	0.041 (2)	0.052 (3)	0.005 (2)	−0.029 (2)	−0.019 (2)
F61	0.041 (3)	0.077 (5)	0.118 (7)	0.014 (3)	0.012 (4)	−0.040 (5)
F62	0.096 (7)	0.077 (5)	0.065 (4)	0.016 (5)	0.023 (3)	−0.038 (3)
F63	0.071 (5)	0.039 (3)	0.084 (6)	0.020 (3)	0.012 (4)	−0.015 (3)
F61'	0.045 (7)	0.082 (10)	0.084 (10)	0.027 (6)	−0.025 (6)	−0.048 (8)
F62'	0.057 (9)	0.052 (7)	0.050 (7)	0.007 (6)	0.033 (5)	−0.013 (5)
F63'	0.061 (8)	0.029 (6)	0.066 (9)	0.008 (5)	0.001 (6)	−0.021 (5)
F71	0.094 (8)	0.079 (7)	0.074 (7)	0.056 (6)	−0.040 (6)	−0.052 (6)
F72	0.033 (3)	0.061 (5)	0.068 (6)	0.009 (3)	0.001 (4)	0.000 (4)
F73	0.044 (6)	0.048 (6)	0.048 (5)	−0.003 (4)	−0.014 (3)	0.007 (4)
F71'	0.087 (7)	0.050 (4)	0.067 (6)	0.030 (4)	−0.036 (5)	−0.033 (4)
F72'	0.060 (5)	0.068 (5)	0.080 (7)	0.038 (4)	0.018 (5)	0.007 (5)
F73'	0.052 (7)	0.049 (5)	0.048 (4)	0.008 (4)	−0.027 (3)	−0.015 (3)
F161	0.070 (5)	0.080 (7)	0.084 (7)	−0.031 (4)	0.018 (5)	−0.061 (6)
F162	0.044 (3)	0.052 (4)	0.074 (6)	−0.004 (3)	−0.031 (3)	−0.004 (3)
F163	0.049 (5)	0.046 (5)	0.048 (4)	−0.002 (4)	−0.010 (3)	0.009 (3)
F16'	0.054 (5)	0.042 (4)	0.060 (6)	−0.006 (3)	0.005 (4)	−0.027 (4)
F16"	0.080 (7)	0.058 (5)	0.083 (8)	−0.026 (5)	−0.052 (7)	0.003 (5)
F16*	0.049 (7)	0.049 (6)	0.047 (5)	−0.006 (4)	0.016 (4)	−0.022 (4)
F171	0.046 (6)	0.090 (10)	0.094 (11)	−0.011 (6)	−0.009 (6)	−0.053 (8)
F172	0.067 (9)	0.030 (6)	0.073 (9)	−0.003 (5)	−0.029 (7)	−0.022 (5)
F173	0.082 (9)	0.069 (8)	0.056 (7)	−0.011 (6)	−0.054 (6)	−0.003 (5)
F17'	0.070 (4)	0.080 (5)	0.126 (7)	0.011 (3)	−0.064 (4)	−0.046 (5)
F17"	0.089 (6)	0.041 (3)	0.088 (6)	−0.010 (3)	−0.048 (5)	−0.013 (3)
F17*	0.126 (7)	0.085 (4)	0.062 (4)	−0.007 (5)	−0.049 (4)	−0.040 (3)

### Geometric parameters (Å, °)

**Table:**

K1—O3	2.711 (3)	C5—C7	1.486 (5)
K1—O1	2.729 (3)	C6—F62'	1.319 (12)
K1—O2	2.738 (3)	C6—F63	1.330 (8)
K1—O3^i^	2.767 (3)	C6—F61	1.336 (8)
K1—F16'	2.923 (9)	C6—F63'	1.347 (12)
K1—F72^i^	2.953 (8)	C6—F62	1.351 (8)
K1—F161	2.992 (10)	C6—F61'	1.382 (11)
K1—F17"^ii^	2.998 (7)	C7—F71	1.304 (8)
K1—F172^ii^	3.004 (14)	C7—F72'	1.309 (8)
K1—F61'^iii^	3.016 (11)	C7—F73	1.321 (10)
K1—F72'^i^	3.078 (14)	C7—F73'	1.336 (10)
K1—F61^iii^	3.196 (14)	C7—F71'	1.401 (9)
K1—H2B	3.08 (4)	C7—F72	1.403 (9)
K1—H3A	3.08 (4)	N11—C15	1.347 (5)
K2—O4^iv^	2.705 (3)	N11—N12	1.359 (4)
K2—O1	2.739 (3)	N12—C13	1.349 (5)
K2—O2	2.745 (3)	C13—C14	1.391 (5)
K2—O4	2.754 (3)	C13—C16	1.486 (5)
K2—F162^i^	2.870 (7)	C14—C15	1.388 (6)
K2—F16"^i^	2.938 (13)	C14—H14	0.9500
K2—F63	2.972 (8)	C15—C17	1.487 (6)
K2—F63'	2.978 (15)	C16—F161	1.312 (7)
K2—F171^iii^	3.026 (12)	C16—F16"	1.319 (9)
K2—F71'^ii^	3.075 (8)	C16—F163	1.325 (9)
K2—F71^ii^	3.199 (13)	C16—F16*	1.326 (10)
K2—F17'^iii^	3.215 (13)	C16—F162	1.383 (7)
K2—H1A	3.05 (4)	C16—F16'	1.396 (8)
K2—H1B	3.08 (4)	C17—F173	1.309 (12)
K2—H4A	3.08 (4)	C17—F17'	1.323 (8)
O1—H1A	0.817 (12)	C17—F17"	1.324 (8)
O1—H1B	0.818 (12)	C17—F172	1.344 (12)
O2—H2A	0.817 (12)	C17—F17*	1.360 (8)
O2—H2B	0.813 (12)	C17—F171	1.401 (11)
O3—K1^i^	2.767 (3)	F61—K1^iii^	3.196 (14)
O3—H3A	0.818 (12)	F61'—K1^iii^	3.016 (11)
O3—H3B	0.819 (12)	F71—K2^v^	3.199 (13)
O4—K2^iv^	2.705 (3)	F72—K1^i^	2.953 (8)
O4—H4A	0.816 (12)	F71'—K2^v^	3.075 (8)
O4—H4B	0.817 (12)	F72'—K1^i^	3.078 (14)
N1—C5	1.348 (5)	F162—K2^i^	2.870 (7)
N1—N2	1.363 (4)	F16"—K2^i^	2.938 (13)
N2—C3	1.349 (5)	F171—K2^iii^	3.026 (12)
C3—C4	1.390 (6)	F172—K1^v^	3.004 (14)
C3—C6	1.480 (6)	F17'—K2^iii^	3.215 (13)
C4—C5	1.390 (5)	F17"—K1^v^	2.998 (7)
C4—H4	0.9500		
			
O3—K1—O1	120.18 (8)	F71'^ii^—K2—F17'^iii^	67.3 (4)
O3—K1—O2	79.20 (8)	F71^ii^—K2—F17'^iii^	78.5 (4)
O1—K1—O2	74.08 (8)	O4^iv^—K2—H1A	110.8 (7)
O3—K1—O3^i^	73.56 (8)	O1—K2—H1A	15.0 (4)
O1—K1—O3^i^	75.98 (7)	O2—K2—H1A	82.4 (7)
O2—K1—O3^i^	120.62 (9)	O4—K2—H1A	60.0 (5)
O3—K1—F16'	78.1 (2)	F162^i^—K2—H1A	90.6 (6)
O1—K1—F16'	147.4 (2)	F16"^i^—K2—H1A	82.5 (6)
O2—K1—F16'	138.4 (2)	F63—K2—H1A	148.9 (6)
O3^i^—K1—F16'	85.3 (4)	F63'—K2—H1A	146.8 (7)
O3—K1—F72^i^	135.5 (3)	F171^iii^—K2—H1A	135.2 (7)
O1—K1—F72^i^	83.5 (4)	F71'^ii^—K2—H1A	82.8 (9)
O2—K1—F72^i^	145.3 (3)	F71^ii^—K2—H1A	69.8 (9)
O3^i^—K1—F72^i^	77.4 (3)	F17'^iii^—K2—H1A	142.1 (7)
F16'—K1—F72^i^	66.4 (4)	O4^iv^—K2—H1B	134.5 (5)
O3—K1—F161	68.8 (3)	O1—K2—H1B	14.7 (5)
O1—K1—F161	142.8 (2)	O2—K2—H1B	81.1 (7)
O2—K1—F161	140.27 (17)	O4—K2—H1B	80.8 (8)
O3^i^—K1—F161	72.6 (4)	F162^i^—K2—H1B	65.2 (6)
F16'—K1—F161	14.2 (2)	F16"^i^—K2—H1B	57.7 (8)
F72^i^—K1—F161	70.7 (4)	F63—K2—H1B	128.9 (8)
O3—K1—F17"^ii^	154.3 (3)	F63'—K2—H1B	123.7 (9)
O1—K1—F17"^ii^	69.4 (3)	F171^iii^—K2—H1B	140.6 (8)
O2—K1—F17"^ii^	81.3 (3)	F71'^ii^—K2—H1B	72.3 (7)
O3^i^—K1—F17"^ii^	131.5 (2)	F71^ii^—K2—H1B	61.4 (7)
F16'—K1—F17"^ii^	106.5 (5)	F17'^iii^—K2—H1B	139.6 (6)
F72^i^—K1—F17"^ii^	66.0 (4)	H1A—K2—H1B	25.4 (8)
F161—K1—F17"^ii^	119.6 (5)	O4^iv^—K2—H4A	81.5 (8)
O3—K1—F172^ii^	157.4 (5)	O1—K2—H4A	60.5 (5)
O1—K1—F172^ii^	74.9 (5)	O2—K2—H4A	110.9 (7)
O2—K1—F172^ii^	90.2 (5)	O4—K2—H4A	14.8 (5)
O3^i^—K1—F172^ii^	128.7 (5)	F162^i^—K2—H4A	125.7 (8)
F16'—K1—F172^ii^	97.7 (6)	F16"^i^—K2—H4A	123.9 (7)
F72^i^—K1—F172^ii^	58.1 (5)	F63—K2—H4A	163.1 (7)
F161—K1—F172^ii^	110.5 (6)	F63'—K2—H4A	167.5 (7)
F17"^ii^—K1—F172^ii^	9.5 (4)	F171^iii^—K2—H4A	93.4 (7)
O3—K1—F61'^iii^	83.0 (6)	F71'^ii^—K2—H4A	79.4 (9)
O1—K1—F61'^iii^	135.1 (3)	F71^ii^—K2—H4A	68.9 (9)
O2—K1—F61'^iii^	73.7 (6)	F17'^iii^—K2—H4A	104.0 (6)
O3^i^—K1—F61'^iii^	148.4 (3)	H1A—K2—H4A	45.5 (7)
F16'—K1—F61'^iii^	69.2 (5)	H1B—K2—H4A	67.6 (9)
F72^i^—K1—F61'^iii^	107.0 (8)	K1—O1—K2	106.24 (9)
F161—K1—F61'^iii^	79.4 (5)	K1—O1—H1A	116 (3)
F17"^ii^—K1—F61'^iii^	75.5 (6)	K2—O1—H1A	105 (3)
F172^ii^—K1—F61'^iii^	74.8 (8)	K1—O1—H1B	110 (3)
O3—K1—F72'^i^	136.4 (2)	K2—O1—H1B	107 (3)
O1—K1—F72'^i^	71.0 (4)	H1A—O1—H1B	111 (4)
O2—K1—F72'^i^	139.7 (2)	K1—O2—K2	105.85 (9)
O3^i^—K1—F72'^i^	68.9 (4)	K1—O2—H2A	113 (3)
F16'—K1—F72'^i^	77.5 (4)	K2—O2—H2A	110 (3)
F72^i^—K1—F72'^i^	14.0 (2)	K1—O2—H2B	107 (3)
F161—K1—F72'^i^	79.4 (3)	K2—O2—H2B	119 (3)
F17"^ii^—K1—F72'^i^	68.2 (4)	H2A—O2—H2B	102 (4)
F172^ii^—K1—F72'^i^	62.0 (6)	K1—O3—K1^i^	106.44 (8)
F61'^iii^—K1—F72'^i^	120.2 (9)	K1—O3—H3A	110 (3)
O3—K1—F61^iii^	92.7 (3)	K1^i^—O3—H3A	103 (3)
O1—K1—F61^iii^	132.49 (19)	K1—O3—H3B	121 (3)
O2—K1—F61^iii^	80.5 (2)	K1^i^—O3—H3B	108 (3)
O3^i^—K1—F61^iii^	150.58 (15)	H3A—O3—H3B	108 (4)
F16'—K1—F61^iii^	66.2 (4)	K2^iv^—O4—K2	105.09 (9)
F72^i^—K1—F61^iii^	96.5 (5)	K2^iv^—O4—H4A	109 (3)
F161—K1—F61^iii^	78.2 (4)	K2—O4—H4A	106 (3)
F17"^ii^—K1—F61^iii^	67.5 (4)	K2^iv^—O4—H4B	116 (3)
F172^ii^—K1—F61^iii^	65.6 (5)	K2—O4—H4B	110 (3)
F61'^iii^—K1—F61^iii^	11.0 (5)	H4A—O4—H4B	111 (5)
F72'^i^—K1—F61^iii^	109.4 (5)	C5—N1—N2	107.6 (3)
O3—K1—H2B	83.5 (8)	C3—N2—N1	107.1 (3)
O1—K1—H2B	83.4 (7)	N2—C3—C4	111.6 (3)
O2—K1—H2B	14.6 (5)	N2—C3—C6	118.8 (4)
O3^i^—K1—H2B	134.9 (5)	C4—C3—C6	129.6 (4)
F16'—K1—H2B	127.6 (7)	C5—C4—C3	102.4 (3)
F72^i^—K1—H2B	139.6 (8)	C5—C4—H4	128.8
F161—K1—H2B	133.5 (8)	C3—C4—H4	128.8
F17"^ii^—K1—H2B	73.7 (8)	N1—C5—C4	111.3 (3)
F172^ii^—K1—H2B	81.7 (9)	N1—C5—C7	119.1 (3)
F61'^iii^—K1—H2B	60.1 (7)	C4—C5—C7	129.6 (4)
F72'^i^—K1—H2B	139.5 (8)	F62'—C6—F63	118.9 (12)
F61^iii^—K1—H2B	66.2 (5)	F62'—C6—F61	82.4 (10)
O3—K1—H3A	14.5 (5)	F63—C6—F61	107.1 (6)
O1—K1—H3A	134.5 (5)	F62'—C6—F63'	105.6 (11)
O2—K1—H3A	87.1 (7)	F63—C6—F62	106.9 (7)
O3^i^—K1—H3A	79.2 (8)	F61—C6—F62	104.9 (6)
F16'—K1—H3A	64.9 (6)	F62'—C6—F61'	104.8 (9)
F72^i^—K1—H3A	127.0 (7)	F63'—C6—F61'	104.6 (10)
F161—K1—H3A	57.1 (7)	F62'—C6—C3	116.1 (11)
F17"^ii^—K1—H3A	148.8 (8)	F63—C6—C3	114.6 (6)
F172^ii^—K1—H3A	147.5 (8)	F61—C6—C3	112.7 (5)
F61'^iii^—K1—H3A	73.4 (9)	F63'—C6—C3	111.6 (12)
F72'^i^—K1—H3A	132.1 (8)	F62—C6—C3	110.0 (6)
F61^iii^—K1—H3A	82.1 (7)	F61'—C6—C3	113.1 (8)
H2B—K1—H3A	88.1 (11)	F71—C7—F73	111.2 (9)
O4^iv^—K2—O1	119.89 (9)	F72'—C7—F73'	110.1 (8)
O4^iv^—K2—O2	79.81 (8)	F72'—C7—F71'	104.3 (7)
O1—K2—O2	73.82 (8)	F73'—C7—F71'	101.9 (7)
O4^iv^—K2—O4	74.91 (9)	F71—C7—F72	104.5 (7)
O1—K2—O4	74.86 (8)	F73—C7—F72	103.4 (7)
O2—K2—O4	121.46 (9)	F71—C7—C5	115.6 (5)
O4^iv^—K2—F162^i^	152.75 (19)	F72'—C7—C5	115.0 (5)
O1—K2—F162^i^	78.0 (2)	F73—C7—C5	111.8 (9)
O2—K2—F162^i^	86.8 (3)	F73'—C7—C5	114.7 (8)
O4—K2—F162^i^	132.0 (2)	F71'—C7—C5	109.6 (5)
O4^iv^—K2—F16"^i^	148.2 (3)	F72—C7—C5	109.3 (6)
O1—K2—F16"^i^	68.5 (4)	C15—N11—N12	107.1 (3)
O2—K2—F16"^i^	73.4 (5)	C13—N12—N11	107.4 (3)
O4—K2—F16"^i^	134.2 (2)	N12—C13—C14	111.5 (3)
F162^i^—K2—F16"^i^	14.9 (3)	N12—C13—C16	119.3 (3)
O4^iv^—K2—F63	82.9 (4)	C14—C13—C16	129.2 (3)
O1—K2—F63	134.4 (2)	C15—C14—C13	102.1 (3)
O2—K2—F63	72.5 (3)	C15—C14—H14	129.0
O4—K2—F63	150.2 (3)	C13—C14—H14	129.0
F162^i^—K2—F63	70.4 (4)	N11—C15—C14	111.9 (3)
F16"^i^—K2—F63	73.0 (4)	N11—C15—C17	118.3 (4)
O4^iv^—K2—F63'	91.8 (6)	C14—C15—C17	129.8 (4)
O1—K2—F63'	131.8 (6)	F161—C16—F16"	74.0 (7)
O2—K2—F63'	78.0 (5)	F161—C16—F163	109.9 (8)
O4—K2—F63'	152.7 (5)	F161—C16—F16*	120.8 (10)
F162^i^—K2—F63'	62.0 (6)	F16"—C16—F16*	110.0 (8)
F16"^i^—K2—F63'	66.3 (7)	F161—C16—F162	104.9 (6)
F63—K2—F63'	9.8 (4)	F163—C16—F162	103.5 (6)
O4^iv^—K2—F171^iii^	70.5 (5)	F16"—C16—F16'	103.7 (7)
O1—K2—F171^iii^	147.0 (3)	F16*—C16—F16'	101.1 (7)
O2—K2—F171^iii^	138.2 (3)	F161—C16—C13	114.8 (5)
O4—K2—F171^iii^	78.8 (6)	F16"—C16—C13	115.6 (5)
F162^i^—K2—F171^iii^	106.5 (7)	F163—C16—C13	112.7 (7)
F16"^i^—K2—F171^iii^	120.8 (9)	F16*—C16—C13	114.9 (9)
F63—K2—F171^iii^	75.2 (5)	F162—C16—C13	110.3 (5)
F63'—K2—F171^iii^	74.3 (7)	F16'—C16—C13	110.0 (5)
O4^iv^—K2—F71'^ii^	135.0 (2)	F17'—C17—F17"	107.7 (6)
O1—K2—F71'^ii^	84.2 (4)	F173—C17—F172	106.8 (12)
O2—K2—F71'^ii^	145.2 (2)	F17'—C17—F17*	104.7 (6)
O4—K2—F71'^ii^	76.3 (2)	F17"—C17—F17*	107.3 (7)
F162^i^—K2—F71'^ii^	62.0 (3)	F173—C17—F171	103.8 (9)
F16"^i^—K2—F71'^ii^	73.6 (4)	F172—C17—F171	103.0 (9)
F63—K2—F71'^ii^	107.6 (5)	F173—C17—C15	118.0 (9)
F63'—K2—F71'^ii^	98.4 (6)	F17'—C17—C15	112.7 (5)
F171^iii^—K2—F71'^ii^	70.4 (5)	F17"—C17—C15	114.7 (6)
O4^iv^—K2—F71^ii^	136.31 (19)	F172—C17—C15	111.1 (11)
O1—K2—F71^ii^	72.3 (4)	F17*—C17—C15	109.0 (6)
O2—K2—F71^ii^	140.0 (2)	F171—C17—C15	112.9 (8)
O4—K2—F71^ii^	68.3 (3)	C6—F61—K1^iii^	139.0 (9)
F162^i^—K2—F71^ii^	66.0 (4)	C6—F63—K2	152.7 (9)
F16"^i^—K2—F71^ii^	75.0 (4)	C6—F61'—K1^iii^	153.2 (11)
F63—K2—F71^ii^	119.9 (6)	C6—F63'—K2	149.8 (16)
F63'—K2—F71^ii^	110.4 (7)	C7—F71—K2^v^	144.1 (11)
F171^iii^—K2—F71^ii^	79.7 (5)	C7—F72—K1^i^	147.6 (8)
F71'^ii^—K2—F71^ii^	13.2 (2)	C7—F71'—K2^v^	147.7 (7)
O4^iv^—K2—F17'^iii^	78.53 (18)	C7—F72'—K1^i^	143.7 (12)
O1—K2—F17'^iii^	150.29 (16)	C16—F161—K1	146.9 (10)
O2—K2—F17'^iii^	135.21 (19)	C16—F162—K2^i^	146.9 (8)
O4—K2—F17'^iii^	89.6 (3)	C16—F16'—K1	145.8 (8)
F162^i^—K2—F17'^iii^	95.1 (4)	C16—F16"—K2^i^	145.9 (13)
F16"^i^—K2—F17'^iii^	109.1 (6)	C17—F171—K2^iii^	151.7 (11)
F63—K2—F17'^iii^	66.3 (4)	C17—F172—K1^v^	151.2 (16)
F63'—K2—F17'^iii^	64.1 (6)	C17—F17'—K2^iii^	139.8 (8)
F171^iii^—K2—F17'^iii^	12.2 (5)	C17—F17"—K1^v^	154.6 (8)
			
O3—K1—O1—K2	−66.68 (11)	F61'—C6—F61—K1^iii^	40 (2)
O2—K1—O1—K2	0.29 (9)	C3—C6—F61—K1^iii^	−56.9 (15)
O3^i^—K1—O1—K2	−127.82 (10)	F62'—C6—F63—K2	119 (3)
F16'—K1—O1—K2	175.4 (6)	F61—C6—F63—K2	−151 (3)
F72^i^—K1—O1—K2	153.5 (2)	F63'—C6—F63—K2	62 (4)
F161—K1—O1—K2	−160.9 (7)	F62—C6—F63—K2	97 (3)
F17"^ii^—K1—O1—K2	86.8 (3)	F61'—C6—F63—K2	−138 (3)
F172^ii^—K1—O1—K2	94.9 (5)	C3—C6—F63—K2	−25 (3)
F61'^iii^—K1—O1—K2	45.9 (10)	O4^iv^—K2—F63—C6	143 (3)
F72'^i^—K1—O1—K2	160.0 (4)	O1—K2—F63—C6	18 (3)
F61^iii^—K1—O1—K2	60.8 (4)	O2—K2—F63—C6	62 (3)
O4^iv^—K2—O1—K1	−68.00 (11)	O4—K2—F63—C6	−175 (2)
O2—K2—O1—K1	−0.29 (9)	F162^i^—K2—F63—C6	−31 (3)
O4—K2—O1—K1	−130.19 (10)	F16"^i^—K2—F63—C6	−16 (3)
F162^i^—K2—O1—K1	89.8 (3)	F63'—K2—F63—C6	−61 (4)
F16"^i^—K2—O1—K1	77.8 (4)	F171^iii^—K2—F63—C6	−145 (3)
F63—K2—O1—K1	43.4 (6)	F71'^ii^—K2—F63—C6	−82 (3)
F63'—K2—O1—K1	56.3 (7)	F71^ii^—K2—F63—C6	−76 (3)
F171^iii^—K2—O1—K1	−168.3 (12)	F17'^iii^—K2—F63—C6	−136 (3)
F71'^ii^—K2—O1—K1	152.4 (2)	F62'—C6—F61'—K1^iii^	−113 (5)
F71^ii^—K2—O1—K1	158.2 (3)	F63—C6—F61'—K1^iii^	128 (5)
F17'^iii^—K2—O1—K1	169.0 (5)	F61—C6—F61'—K1^iii^	−81 (5)
O3—K1—O2—K2	125.63 (10)	F63'—C6—F61'—K1^iii^	136 (5)
O1—K1—O2—K2	−0.29 (9)	F62—C6—F61'—K1^iii^	−125 (5)
O3^i^—K1—O2—K2	62.23 (12)	C3—C6—F61'—K1^iii^	14 (5)
F16'—K1—O2—K2	−176.3 (5)	F62'—C6—F63'—K2	176 (4)
F72^i^—K1—O2—K2	−52.0 (7)	F63—C6—F63'—K2	−54 (3)
F161—K1—O2—K2	162.0 (7)	F61—C6—F63'—K2	−93 (4)
F17"^ii^—K1—O2—K2	−71.3 (3)	F62—C6—F63'—K2	160 (4)
F172^ii^—K1—O2—K2	−74.5 (5)	F61'—C6—F63'—K2	−74 (4)
F61'^iii^—K1—O2—K2	−148.6 (6)	C3—C6—F63'—K2	49 (4)
F72'^i^—K1—O2—K2	−30.8 (7)	O4^iv^—K2—F63'—C6	76 (4)
F61^iii^—K1—O2—K2	−139.7 (3)	O1—K2—F63'—C6	−58 (4)
O4^iv^—K2—O2—K1	125.69 (10)	O2—K2—F63'—C6	−3(4)
O1—K2—O2—K1	0.29 (9)	O4—K2—F63'—C6	136 (3)
O4—K2—O2—K1	60.53 (12)	F162^i^—K2—F63'—C6	−95 (4)
F162^i^—K2—O2—K1	−78.2 (2)	F16"^i^—K2—F63'—C6	−80 (4)
F16"^i^—K2—O2—K1	−71.6 (4)	F63—K2—F63'—C6	52 (3)
F63—K2—O2—K1	−148.6 (4)	F171^iii^—K2—F63'—C6	146 (4)
F63'—K2—O2—K1	−140.2 (6)	F71'^ii^—K2—F63'—C6	−148 (4)
F171^iii^—K2—O2—K1	170.5 (10)	F71^ii^—K2—F63'—C6	−142 (3)
F71'^ii^—K2—O2—K1	−52.8 (6)	F17'^iii^—K2—F63'—C6	153 (4)
F71^ii^—K2—O2—K1	−32.7 (7)	F72'—C7—F71—K2^v^	−150 (3)
F17'^iii^—K2—O2—K1	−172.2 (3)	F73—C7—F71—K2^v^	90 (2)
O1—K1—O3—K1^i^	−62.37 (11)	F73'—C7—F71—K2^v^	106 (2)
O2—K1—O3—K1^i^	−126.65 (10)	F71'—C7—F71—K2^v^	46.7 (18)
O3^i^—K1—O3—K1^i^	0.0	F72—C7—F71—K2^v^	−159 (2)
F16'—K1—O3—K1^i^	88.5 (3)	C5—C7—F71—K2^v^	−39 (3)
F72^i^—K1—O3—K1^i^	51.4 (5)	F71—C7—F72—K1^i^	73 (2)
F161—K1—O3—K1^i^	77.3 (4)	F72'—C7—F72—K1^i^	55.2 (18)
F17"^ii^—K1—O3—K1^i^	−168.1 (7)	F73—C7—F72—K1^i^	−171 (2)
F172^ii^—K1—O3—K1^i^	169.9 (13)	F73'—C7—F72—K1^i^	−166 (2)
F61'^iii^—K1—O3—K1^i^	158.6 (5)	F71'—C7—F72—K1^i^	90 (2)
F72'^i^—K1—O3—K1^i^	31.3 (7)	C5—C7—F72—K1^i^	−51 (2)
F61^iii^—K1—O3—K1^i^	153.52 (18)	F71—C7—F71'—K2^v^	−56.2 (17)
O4^iv^—K2—O4—K2^iv^	0.0	F72'—C7—F71'—K2^v^	−72 (2)
O1—K2—O4—K2^iv^	127.42 (11)	F73—C7—F71'—K2^v^	164 (2)
O2—K2—O4—K2^iv^	67.68 (11)	F73'—C7—F71'—K2^v^	173 (2)
F162^i^—K2—O4—K2^iv^	−174.8 (4)	F72—C7—F71'—K2^v^	−91 (2)
F16"^i^—K2—O4—K2^iv^	165.0 (7)	C5—C7—F71'—K2^v^	51 (2)
F63—K2—O4—K2^iv^	−43.3 (8)	F71—C7—F72'—K1^i^	152 (3)
F63'—K2—O4—K2^iv^	−63.2 (12)	F73—C7—F72'—K1^i^	−102 (2)
F171^iii^—K2—O4—K2^iv^	−72.7 (5)	F73'—C7—F72'—K1^i^	−91 (2)
F71'^ii^—K2—O4—K2^iv^	−145.0 (4)	F71'—C7—F72'—K1^i^	161 (2)
F71^ii^—K2—O4—K2^iv^	−156.0 (4)	F72—C7—F72'—K1^i^	−45.6 (19)
F17'^iii^—K2—O4—K2^iv^	−78.21 (16)	C5—C7—F72'—K1^i^	41 (3)
C5—N1—N2—C3	−0.2 (4)	F16"—C16—F161—K1	−150 (3)
N1—N2—C3—C4	0.7 (4)	F163—C16—F161—K1	89 (2)
N1—N2—C3—C6	−179.3 (3)	F16*—C16—F161—K1	106 (2)
N2—C3—C4—C5	−0.9 (4)	F162—C16—F161—K1	−160 (2)
C6—C3—C4—C5	179.1 (4)	F16'—C16—F161—K1	49 (2)
N2—N1—C5—C4	−0.4 (4)	C13—C16—F161—K1	−39 (3)
N2—N1—C5—C7	179.5 (3)	O3—K1—F161—C16	79 (2)
C3—C4—C5—N1	0.8 (4)	O1—K1—F161—C16	−168.4 (17)
C3—C4—C5—C7	−179.2 (4)	O2—K1—F161—C16	41 (3)
N2—C3—C6—F62'	166.4 (16)	O3^i^—K1—F161—C16	158 (2)
C4—C3—C6—F62'	−13.6 (17)	F16'—K1—F161—C16	−50.1 (19)
N2—C3—C6—F63	−49.0 (12)	F72^i^—K1—F161—C16	−120 (2)
C4—C3—C6—F63	131.0 (11)	F17"^ii^—K1—F161—C16	−74 (2)
N2—C3—C6—F61	73.8 (10)	F172^ii^—K1—F161—C16	−77 (2)
C4—C3—C6—F61	−106.2 (11)	F61'^iii^—K1—F161—C16	−7(2)
N2—C3—C6—F63'	−72.5 (15)	F72'^i^—K1—F161—C16	−131 (2)
C4—C3—C6—F63'	107.5 (14)	F61^iii^—K1—F161—C16	−18 (2)
N2—C3—C6—F62	−169.4 (10)	F161—C16—F162—K2^i^	67 (2)
C4—C3—C6—F62	10.6 (11)	F16"—C16—F162—K2^i^	48.6 (15)
N2—C3—C6—F61'	45.1 (18)	F163—C16—F162—K2^i^	−178.1 (18)
C4—C3—C6—F61'	−134.9 (18)	F16*—C16—F162—K2^i^	−172.5 (16)
N1—C5—C7—F71	−37.7 (14)	F16'—C16—F162—K2^i^	86.4 (18)
C4—C5—C7—F71	142.3 (14)	C13—C16—F162—K2^i^	−57.4 (17)
N1—C5—C7—F72'	46.2 (14)	F161—C16—F16'—K1	−49.2 (15)
C4—C5—C7—F72'	−133.9 (14)	F16"—C16—F16'—K1	−68 (2)
N1—C5—C7—F73	−166.2 (10)	F163—C16—F16'—K1	168.2 (18)
C4—C5—C7—F73	13.7 (11)	F16*—C16—F16'—K1	178 (2)
N1—C5—C7—F73'	175.3 (9)	F162—C16—F16'—K1	−88.0 (19)
C4—C5—C7—F73'	−4.7 (10)	C13—C16—F16'—K1	55.9 (19)
N1—C5—C7—F71'	−70.8 (9)	O3—K1—F16'—C16	−2.9 (16)
C4—C5—C7—F71'	109.1 (9)	O1—K1—F16'—C16	125.8 (13)
N1—C5—C7—F72	79.8 (9)	O2—K1—F16'—C16	−61 (2)
C4—C5—C7—F72	−100.2 (10)	O3^i^—K1—F16'—C16	71.3 (17)
C15—N11—N12—C13	−0.1 (4)	F72^i^—K1—F16'—C16	149.6 (19)
N11—N12—C13—C14	−0.4 (4)	F161—K1—F16'—C16	44.6 (13)
N11—N12—C13—C16	179.1 (3)	F17"^ii^—K1—F16'—C16	−156.8 (17)
N12—C13—C14—C15	0.7 (4)	F172^ii^—K1—F16'—C16	−160.3 (18)
C16—C13—C14—C15	−178.7 (4)	F61'^iii^—K1—F16'—C16	−89.8 (18)
N12—N11—C15—C14	0.6 (4)	F72'^i^—K1—F16'—C16	140.7 (18)
N12—N11—C15—C17	−178.8 (3)	F61^iii^—K1—F16'—C16	−101.3 (17)
C13—C14—C15—N11	−0.8 (4)	F161—C16—F16"—K2^i^	153 (3)
C13—C14—C15—C17	178.5 (4)	F163—C16—F16"—K2^i^	−104 (2)
N12—C13—C16—F161	−40.9 (13)	F16*—C16—F16"—K2^i^	−90 (2)
C14—C13—C16—F161	138.5 (12)	F162—C16—F16"—K2^i^	−46 (2)
N12—C13—C16—F16"	42.5 (15)	F16'—C16—F16"—K2^i^	163 (2)
C14—C13—C16—F16"	−138.1 (15)	C13—C16—F16"—K2^i^	42 (3)
N12—C13—C16—F163	−167.7 (8)	F173—C17—F171—K2^iii^	−104 (4)
C14—C13—C16—F163	11.8 (9)	F17'—C17—F171—K2^iii^	−71 (4)
N12—C13—C16—F16*	172.3 (9)	F17"—C17—F171—K2^iii^	139 (4)
C14—C13—C16—F16*	−8.2 (11)	F172—C17—F171—K2^iii^	145 (4)
N12—C13—C16—F162	77.3 (8)	F17*—C17—F171—K2^iii^	−115 (4)
C14—C13—C16—F162	−103.3 (8)	C15—C17—F171—K2^iii^	25 (5)
N12—C13—C16—F16'	−74.5 (9)	F173—C17—F172—K1^v^	−177 (3)
C14—C13—C16—F16'	105.0 (9)	F17'—C17—F172—K1^v^	−89 (4)
N11—C15—C17—F173	165.3 (15)	F17"—C17—F172—K1^v^	−51 (3)
C14—C15—C17—F173	−14.0 (16)	F17*—C17—F172—K1^v^	163 (3)
N11—C15—C17—F17'	75.8 (9)	F171—C17—F172—K1^v^	−68 (4)
C14—C15—C17—F17'	−103.5 (9)	C15—C17—F172—K1^v^	53 (3)
N11—C15—C17—F17"	−48.0 (10)	F173—C17—F17'—K2^iii^	−171.6 (16)
C14—C15—C17—F17"	132.7 (9)	F17"—C17—F17'—K2^iii^	71.9 (14)
N11—C15—C17—F172	−70.9 (13)	F172—C17—F17'—K2^iii^	85.4 (18)
C14—C15—C17—F172	109.8 (13)	F17*—C17—F17'—K2^iii^	−174.0 (10)
N11—C15—C17—F17*	−168.3 (8)	F171—C17—F17'—K2^iii^	40.8 (17)
C14—C15—C17—F17*	12.3 (9)	C15—C17—F17'—K2^iii^	−55.7 (14)
N11—C15—C17—F171	44.2 (16)	F173—C17—F17"—K1^v^	123 (3)
C14—C15—C17—F171	−135.2 (16)	F17'—C17—F17"—K1^v^	−150 (2)
F62'—C6—F61—K1^iii^	−172.1 (17)	F172—C17—F17"—K1^v^	62 (4)
F63—C6—F61—K1^iii^	70.0 (16)	F17*—C17—F17"—K1^v^	98 (3)
F63'—C6—F61—K1^iii^	84.2 (19)	F171—C17—F17"—K1^v^	−135 (3)
F62—C6—F61—K1^iii^	−176.6 (12)	C15—C17—F17"—K1^v^	−23 (3)

Symmetry codes: (i) −*x*+1, −*y*+1, −*z*+1; (ii) *x*, *y*+1, *z*; (iii) −*x*, −*y*+1, −*z*+1; (iv) −*x*, −*y*+2, −*z*+1; (v) *x*, *y*−1, *z*.

### Hydrogen-bond geometry (Å, °)

**Table:**

*D*—H···*A*	*D*—H	H···*A*	*D*···*A*	*D*—H···*A*
O1—H1A···N11^ii^	0.82 (2)	2.10 (2)	2.906 (4)	167 (4)
O1—H1B···N12^i^	0.82 (2)	2.09 (2)	2.896 (4)	168 (4)
O2—H2A···N2	0.82 (2)	2.12 (1)	2.929 (4)	173 (5)
O2—H2B···N2^iii^	0.81 (2)	2.07 (1)	2.879 (4)	173 (5)
O3—H3A···N12	0.82 (2)	2.05 (1)	2.868 (4)	173 (4)
O3—H3B···N1	0.82 (2)	2.09 (1)	2.910 (4)	177 (4)
O4—H4A···N11^ii^	0.82 (2)	2.08 (1)	2.891 (4)	175 (4)
O4—H4B···N1^ii^	0.82 (2)	2.07 (1)	2.884 (4)	174 (4)

Symmetry codes: (ii) *x*, *y*+1, *z*; (i) −*x*+1, −*y*+1, −*z*+1; (iii) −*x*, −*y*+1, −*z*+1.
